# Supplementary material for: Increased public health threat of avian-origin H3N2 influenza virus caused by its evolution in dogs
Source: eLife. 2023 Apr 6;12:e83470. doi: 10.7554/eLife.83470 (PMC10147381; doi:10.7554/eLife.83470)
Supplement: Supplementary file 1. — HI titers are the inverse of the highest dilution that inhibited hemagglutination. Cells containing moderate HI titers (160, 320) are shaded gray, and high titers (>640) black. Low HI titers (40, 80) are not shaded. Titers <10 are indicated with the sign <. HI, hemagglutinin inhibition. †Homologous titer. [file elife-83470-supp1.docx]

**Table S1. Antigenic analysis of H3N2 subtype canine influenza viruses in the world**

|  | HI titer, by antigenic group and antiserum | | | | | | | | | | | | | |
| --- | --- | --- | --- | --- | --- | --- | --- | --- | --- | --- | --- | --- | --- | --- |
| Antigenic group and virus (clade) | Human,BJ/1230/16 | A,  Cn/GD/1/06 | A,  Cn/KR/01/07 | B,  Cn/BJ/358/09 | B,  Cn/BJ/362/09 | C,  Cn/BJ/256/15 | C,  Cn/BJ/265/15 | D,  Cn/KR/AS-03/12 | D,  Cn/IL/M17-05782-7-1/17 | E,  Cn/BJ/137/17 | E,  Cn/BJ/147/17 | F,  Cn/FJ/1109/18 | G,  Cn/GZ/011/19 | G,  Cn/BJ/1115/19 |
| BJ/1230/16  (Human) | **1280†** | 40 | 20 | 40 | 40 | 160 | 160 | 20 | 20 | 20 | 20 | 80 | 160 | 160 |
| A |  |  |  |  |  |  |  |  |  |  |  |  |  |  |
| Cn/GD/1/06  (clade0) | < | **1280†** | 640 | 80 | 80 | 40 | 40 | < | < | 160 | 160 | 40 | 80 | 160 |
| Cn/KR/01/07  (clade1) | < | 1280 | **1280†** | 40 | 40 | 40 | 40 | < | < | 160 | 160 | 40 | 40 | 80 |
| B |  |  |  |  |  |  |  |  |  |  |  |  |  |  |
| Cn/BJ/358/09  (clade2) | < | 320 | 160 | **1280†** | 1280 | 160 | 320 | < | < | 320 | 160 | 80 | 160 | 160 |
| Cn/BJ/362/09  (clade2) | < | 320 | 160 | 1280 | **1280†** | 320 | 160 | < | < | 160 | 160 | 160 | 160 | 160 |
| C |  |  |  |  |  |  |  |  |  |  |  |  |  |  |
| Cn/TH/CU-DC5299/12 (clade2) | < | 320 | 160 | 320 | 320 | 1280 | 1280 | < | < | 320 | 160 | 80 | 80 | 80 |
| Cn/BJ/34/12  (clade2) | < | 320 | 320 | 160 | 320 | 1280 | 1280 | < | < | 320 | 320 | 80 | 80 | 80 |
| Cn/BJ/365/13  (clade2) | < | 320 | 640 | 160 | 160 | 1280 | 1280 | < | < | 320 | 320 | 80 | 160 | 80 |
| Cn/BJ/256/15  (clade2) | < | 160 | 160 | 320 | 320 | **1280†** | 1280 | < | < | 160 | 160 | 160 | 80 | 160 |
| Cn/BJ/265/15  (clade2) | < | 640 | 320 | 160 | 160 | 1280 | **1280†** | < | < | 160 | 320 | 80 | 160 | 160 |
| D |  |  |  |  |  |  |  |  |  |  |  |  |  |  |
| Cn/KR/AS-03/12(clade3) | < | 20 | < | < | < | < | < | **1280†** | 1280 | < | < | < | < | < |
| Cn/IL/M17-05782-7-1/17  (clade4) | < | 20 | < | < | < | < | < | 1280 | **1280†** | < | < | < | < | < |
| E |  |  |  |  |  |  |  |  |  |  |  |  |  |  |
| Cn/BJ/38/16  (clade5) | < | 320 | 320 | 80 | 80 | 160 | 320 | < | < | 640 | 640 | 80 | 80 | 80 |
| Cn/BJ/137/17  (clade5) | < | 320 | 320 | 80 | 40 | 160 | 320 | < | < | **1280†** | 1280 | 80 | 80 | 80 |
| Cn/BJ/147/17  (clade5) | < | 160 | 160 | 80 | 80 | 160 | 320 | < | < | 1280 | **1280†** | 80 | 160 | 80 |
| Cn/BJ/308/17  (clade5) | < | 320 | 160 | 80 | 80 | 160 | 320 | < | < | 1280 | 640 | 320 | 320 | 160 |
| Cn/SH/354/17  (clade5) | < | 80 | 160 | 80 | 40 | 160 | 320 | < | < | 640 | 640 | 160 | 320 | 160 |
| Cn/NJ/404/17  (clade5) | < | 320 | 320 | 80 | 80 | 320 | 320 | < | < | 1280 | 640 | 320 | 160 | 320 |
| Cn/BJ/509/17  (clade5) | < | 160 | 80 | 40 | 40 | 160 | 320 | < | < | 640 | 640 | 160 | 320 | 320 |
| Cn/CA/BRW003/18 (clade5) | < | 160 | 320 | 40 | 40 | 40 | 40 | < | < | 640 | 640 | 80 | 320 | 320 |
| Cn/LN/530/18  (clade5) | < | 320 | 320 | 40 | 40 | 160 | 320 | < | < | 640 | 640 | 160 | 320 | 320 |
| Cn/FJ/601/18  (clade5) | < | 160 | 80 | 80 | 40 | 160 | 320 | < | < | 1280 | 1280 | 320 | 160 | 320 |
| Cn/BJ/616/18  (clade5) | < | 320 | 320 | 80 | 80 | 320 | 320 | < | < | 1280 | 640 | 320 | 320 | 320 |
| Cn/TJ/643/18  (clade5) | < | 320 | 160 | 40 | 40 | 320 | 320 | < | < | 1280 | 640 | 320 | 320 | 160 |
| Cn/SX/676/18  (clade5) | < | 320 | 160 | 80 | 80 | 160 | 320 | < | < | 640 | 640 | 160 | 160 | 160 |
| Cn/SH/755/18  (clade5) | < | 160 | 320 | 40 | 40 | 160 | 320 | < | < | 640 | 640 | 160 | 320 | 160 |
| Cn/BJ/85/18  (clade5) | < | 160 | 160 | 80 | 80 | 160 | 320 | < | < | 640 | 640 | 160 | 160 | 160 |
| Cn/BJ/1016/18  (clade5) | < | 160 | 320 | 80 | 80 | 320 | 320 | < | < | 1280 | 1280 | 320 | 320 | 320 |
| Cn/FJ/11/18  (clade5) | < | 160 | 160 | 80 | 80 | 320 | 320 | < | < | 1280 | 1280 | 320 | 320 | 320 |
| Cn/TJ/19/18  (clade5) | < | 160 | 320 | 80 | 40 | 160 | 320 | < | < | 640 | 640 | 160 | 160 | 320 |
| Cn/HaiN/F4/18  (clade5) | < | 320 | 320 | 80 | 80 | 320 | 320 | < | < | 1280 | 1280 | 320 | 320 | 320 |
| Cn/GZ/1180/19  (clade5.1) | < | 320 | 320 | 80 | 80 | 320 | 320 | < | < | 1280 | 1280 | 320 | 320 | 320 |
| Cn/BJ/1183/19  (clade5.1) | < | 320 | 160 | 40 | 40 | 320 | 320 | < | < | 1280 | 1280 | 320 | 320 | 160 |
| Cn/HaiN/079/19(clade5.1) | < | 320 | 320 | 80 | 40 | 320 | 320 | < | < | 1280 | 1280 | 160 | 160 | 320 |
| Cn/BJ/1364/19  (clade5.1) | < | 320 | 160 | 80 | 80 | 320 | 320 | < | < | 1280 | 1280 | 320 | 320 | 320 |
| Cn/SH/1396/19  (clade5.1) | < | 320 | 320 | 80 | 80 | 320 | 320 | < | < | 1280 | 1280 | 160 | 160 | 160 |
| F |  |  |  |  |  |  |  |  |  |  |  |  |  |  |
| Cn/FJ/1108/18  (clade5) | < | 160 | 80 | 20 | 20 | 80 | 160 | < | < | 80 | 160 | 1280 | 320 | 160 |
| Cn/FJ/1109/18  (clade5) | < | 160 | 80 | 40 | 40 | 160 | 80 | < | < | 80 | 80 | **1280†** | 160 | 160 |
| G |  |  |  |  |  |  |  |  |  |  |  |  |  |  |
| Cn/GZ/011/19  (clade5) | < | 320 | 160 | 80 | 40 | 160 | 320 | < | < | 160 | 160 | 160 | **1280†** | 1280 |
| Cn/BJ/1115/19  (clade5) | < | 320 | 160 | 80 | 80 | 160 | 160 | < | < | 160 | 320 | 160 | 1280 | **1280†** |

HI titers are the inverse of the highest dilution that inhibited hemagglutination. Cells containing moderate HI titers (160, 320) are shaded gray and high titers (>640) black. Low HI titers (40, 80) are not shaded. Titers < 10 are indicated with the sign <. HI, hemagglutinin inhibition.

†Homologous titer.
