# Supplementary material for: Increased public health threat of avian-origin H3N2 influenza virus caused by its evolution in dogs
Source: eLife. 2023 Apr 6;12:e83470. doi: 10.7554/eLife.83470 (PMC10147381; doi:10.7554/eLife.83470)
Supplement: Supplementary file 2. — NI titers are the inverse of the highest dilution that inhibited neuraminidase activity. NI, neuraminidase inhibition. †Homologous titer. [file elife-83470-supp2.docx]

**Table S2. NI titers of ferret antisera against human influenza A (H3N2) and H3N2 canine influenza preponderant prevalent viruses used in this study**

|  | Ferret antisera against | | | |
| --- | --- | --- | --- | --- |
| Antigen or virus | BJ/1230/16 (human) | Cn/BJ/38/16 | Cn/FJ/1109/18 | Cn/GZ/011/19 |
| BJ/1230/16 (human) | **2560†** | <10 | <10 | <10 |
| Cn/BJ/38/16 | <10 | **2560†** | 1280 | 2560 |
| Cn/FJ/1109/18 | <10 | 1280 | **2560†** | 1280 |
| Cn/GZ/011/19 | <10 | 2560 | 1280 | **2560†** |

NI titers are the inverse of the highest dilution that inhibited neuraminidase activity. NI, neuraminidase inhibition.†Homologous titer.
