# Supplementary material for: Increased public health threat of avian-origin H3N2 influenza virus caused by its evolution in dogs
Source: eLife. 2023 Apr 6;12:e83470. doi: 10.7554/eLife.83470 (PMC10147381; doi:10.7554/eLife.83470)
Supplement: Supplementary file 4. — aSera were collected from the ferrets 3 weeks after virus inoculation or exposure; these animals were used for the transmission studies shown in Figure 5. HI, hemagglutinin inhibition. [file elife-83470-supp4.docx]

**Table S4. Seroconversion of the ferrets in transmission experiments.**

| Virus | Seroconversion: positive/total (HI titers)^a^ | |
| --- | --- | --- |
|  | Inoculated | Aerosol |
| A/duck/Korea/JS53/2004 | 3/3(80,80,40) | 0/3 |
| A/canine/Guangdong/1/2006 (clade0) | 3/3(160,80,80) | 0/3 |
| A/canine/Korea/01/2007 (clade1) | 3/3(80,80,80) | 0/3 |
| A/canine/Thailand/CU-DC5299/2012 (clade2) | 3/3(160,160,160) | 0/3 |
| A/canine/Beijing/0118-256/2015 (clade2) | 3/3(320,160,160) | 0/3 |
| A/canine/Korea/AS-03/2012 (clade3) | 3/3(320,160,320) | 0/3 |
| A/canine/lllinoins/M17-05782-7-1/2017 (clade4) | 3/3(320,320,320) | 0/3 |
| A/canine/Beijing/1216-38/2016 (clade5) | 3/3(640,320,640) | 3/3(160,320,640) |
| A/canine/Shanghai/159/2017 (clade5) | 3/3(320,320,640) | 3/3(160,160,320) |
| A/canine/California/BRW003/2018 (clade5) | 3/3(320,320,320) | 3/3(160,160,320) |
| A/canine/Hainan/079/2019 (clade5.1) | 3/3(320,640,640) | 3/3(320,320,320) |
| A/canine/Guangzhou/1180/2019 (clade5.1) | 3/3(640,640,320) | 3/3(320,640,320) |
| A/Beijing/1230/2016 (human) | 3/3(640,640,320) | 3/3(640,640,320) |

^a^Sera were collected from the ferrets 3 weeks after virus inoculation or exposure; these animals were used for the transmission studies shown in Fig 5. HI, hemagglutinin inhibition.
