# Supplementary material for: Increased public health threat of avian-origin H3N2 influenza virus caused by its evolution in dogs
Source: eLife. 2023 Apr 6;12:e83470. doi: 10.7554/eLife.83470 (PMC10147381; doi:10.7554/eLife.83470)
Supplement: Supplementary file 6. — ‡Number of animals in which virus was detected in nasal washes. Values in parentheses indicate nasal wash titers, which are expressed as mean log10 peak virus titer observed within the first 9 dpi. §Number of animals in which seroconversion was observed. Values in parentheses show HI antibody titers in sera collected at 21 dpi. HI titers were determined using the homologous virus as a test antigen. [file elife-83470-supp6.docx]

**Table S6. Transmission of H3N2 reassortant viruses in ferrets**

|  | Inoculated Animals | | Aerosol Animals | |
| --- | --- | --- | --- | --- |
| Virus | No. with virus  detection  (peak titer mean log_10_EID_50_/ml)‡ | No. with seroconversion  (HI titer range)§ | No. with virus detection  (peak titer mean log_10_EID_50_/ml)‡ | No. with seroconversion  (HI titer range)§ |
| BJ15 | 3/3(5.75) | 3/3(320-640) | 0/3 | 0/3 |
| HaiN/19 | 3/3(7.84) | 3/3(640-1280) | 3/3(5.75) | 3/3(320-640) |
| rgHaiN19PB2 | 3/3(3.75) | 3/3(320-640) | 0/3 | 0/3 |
| rgHaiN19PB1 | 3/3(6.83) | 3/3(640) | 2/3(4.25) | 2/3(160) |
| rgHaiN19PA | 3/3(4.75) | 3/3(320-640) | 0/3 | 0/3 |
| rgHaiN19HA | 3/3(7.25) | 3/3(160-640) | 2/3(4.75) | 2/3(80-160) |
| rgHaiN19NP | 3/3(6.15) | 3/3(160-320) | 1/3(2.75) | 1/3(40) |
| rgHaiN19NA | 3/3(4.75) | 3/3(160-320) | 0/3 | 0/3 |
| rgHaiN19M | 3/3(4.5) | 3/3(160-320) | 0/3 | 0/3 |
| rgHaiN19NS | 3/3(4.25) | 3/3(160-320) | 0/3 | 0/3 |

‡Number of animals in which virus was detected in nasal washes. Values in parentheses indicate nasal wash titers, which are expressed as mean log_10_ peak virus titer observed within the first 9 dpi.

§Number of animals in which seroconversion was observed. Values in parentheses show HI antibody titers in sera collected at 21 dpi. HI titers were determined using the homologous virus as test antigen.
